# High viral loads of circulating Epstein-Barr virus DNA copy number in peripheral blood is associated with inferior prognosis in patients with mantle cell lymphoma

**DOI:** 10.7150/jca.37484

**Published:** 2020-06-21

**Authors:** Xiao-Hui Zhou, Jin-Hua Liang, Li Wang, Hua-Yuan Zhu, Jia-Zhu Wu, Yi Xia, Yue Li, Shu-Chao Qin, Lei Fan, Jian-Yong Li, Wei Xu

**Affiliations:** 1 Department of Hematology, the First Affiliated Hospital of Nanjing Medical University, Jiangsu Province Hospital, Nanjing 210029, China.; 2 Key Laboratory of Hematology of Nanjing Medical University, Nanjing 210029, China.; 3 Collaborative Innovation Center for Cancer Personalized Medicine, Nanjing 210029, China.

**Keywords:** Mantle cell lymphoma, Circulating Epstein-Barr virus DNA load, Overall survival, Progression free survival

## Abstract

Mantle cell lymphoma (MCL) is a distinct subtype of B cell non-Hodgkin lymphoma. No research has yet documented to investigate the prognostic implications of Epstein-Barr virus (EBV) infection in MCL. The objective of this study was to determine whether EBV DNA load may influence the heterogeneity in the course of the disease in MCL patients. Eighty-eight MCL patients were retrospectively enrolled in the study. EBV DNA load was detected by real-time quantitative PCR for quantification. The univariate and multivariate Cox proportional hazards models were established for the estimation of prognostic factors. Twenty-seven patients were detected positive for EBV DNA and the median virus titer was 1.72×10^4^ copies/mL (range, 8.20×10^2^ to 4.14×10^5^ copies/mL). With a median follow-up of 39 months (range, 9 to 120 months), patients in EBV DNA-positive group displayed unfavorable progression-free survival (PFS) (*P*=0.012) and overall survival (OS) (*P*=0.004) than patients in EBV DNA-negative group. Multivariate Cox regression analysis revealed that EBV DNA-positivity was an independent risk factor for both PFS (HR, 2.04; 95% CI, 1.07 to 3.92; *P*=0.031) and OS (HR, 2.68; 95% CI, 1.20 to 6.00; *P*=0.016). Reduction in EBV copies was significantly associated with therapy-response.

Circulating EBV DNA load in whole blood proved to be a significant predictor of prognosis in patients with MCL, which needs further validation in large-scale clinical studies.

## Introduction

Mantle cell lymphoma (MCL) is a distinct subtype of B cell non-Hodgkin lymphoma (NHL) manifested by extensive lymphadenopathy, blood and bone marrow involvement, and splenomegaly with a short remission duration to standard therapies 1. The past few decades have witnessed great progresses in improving the outcomes of MCL patients owing to the widespread use of rituximab in combination with anthracycline-containing regimens as well as new approaches 2. However, patients with MCL still presented great heterogeneity in the clinical course with a median overall survival (OS) of 3-5 years 1.

Epstein-Barr virus (EBV), also known as human herpesvirus 4 (HHV-4), is a ubiquitous herpesvirus that infects more than 90% of all humans, contributing to the development of EBV-associated lymphomas, lymphoproliferative disorders, hemophagocytic lymphohistiocytosis and solid tumors 3. Several researches showed that high level of EBV DNA loads in whole blood or plasma predicted adverse prognosis in EBV-negative lymphomas, such as chronic lymphocytic leukemia (CLL), invariably EBV-positive lymphomas, such as extranodal natural killer/T-cell lymphoma (ENKTL) and angioimmunoblastic T-cell lymphoma (AITL), and partially EBV-positive lymphomas, such as diffuse large B-cell lymphoma (DLBCL) and Hodgkin lymphoma (HL) 4-8. Though MCL is not currently considered as a subtype of EBV-associated lymphoma, some of the patients are inevitably infected with EBV 9. Notably, due to the rarity of MCL, the pathogenetic roles and detailed mechanisms of EBV in the development of MCL are dismal. Furthermore, there are no reports on the prevalence and clinical significances of EBV in patients with MCL until now. Hence, we conducted the retrospective analysis to comprehensively explore the potential prognostic impacts of circulating EBV DNA load in 88 MCL patients.

## Materials and Methods

### Patients

A total of 88 consecutive subjects histologically newly diagnosed MCL patients between September 2008 and November 2017 were enrolled in this retrospective study in the First Affiliated Hospital of Nanjing Medical University, Jiangsu Province Hospital (Nanjing, China). Diagnosis of MCL, dependent on a surgical specimen, preferably a lymph node biopsy, was in accordance with criteria of World Health Organization (WHO) classification 2008 10. Besides, detection of t(11;14)(q13;q32) and overexpression of cyclin D1 is mandatory for the diagnosis of MCL. Inclusion criteria were as follows: histologically confirmed MCL; at least had one efficacy assessment after receiving first-line chemotherapy of R-hyper-CVAD regimen (rituximab, cyclophosphamide, doxorubicin, vincristine and dexamethasone) alternated with the MA regimen (high-dose methotrexate and cytarabine) or a modified R-hyper-CVAD regimen or R-CHOP-like (rituximab, cyclophosphamide, vincristine, doxorubicin and prednisolone) regimen. Blastoid type of MCL was excluded from this study.

Efficacy assessment was strictly in accordance with International Working Group Criteria and responses were classified as complete response (CR), unconfirmed CR (CRu), partial response (PR), stable disease (SD), and progressive disease (PD) 11.

### Data collection

Baseline clinical characteristics including gender, age, bone marrow involvement (BMI), Ann Arbor stage, Eastern Cooperative Oncology Group (ECOG) score, B symptoms, white blood cell (WBC) counts, absolute monocyte counts (AMC), serum lactic dehydrogenase (LDH), serum beta-2 microglobulin level (β2-MG) and simplified MCL International Prognostic Index (sMIPI) score (covering age, ECOG, LDH and WBC) at admission were entirely available.

EBV DNA load at initial diagnosis, as well as, before each cycle of chemotherapy was detected by real-time quantitative PCR with an ABI PRISM 7500 (Applied Biosystems, Foster City, CA) using DNA extracted with the EBV-PCR Fluorescence Quantitative Diagnostic Kit (Da An Gene Co., Guangzhou, China) from whole blood according to instruction manuals. As for EBV DNA copy number, with the improvement of sensitivity, the boundary of 5×10^2^ copies/mL was established as a cut-off value to distinguish EBV DNA-positive from EBV DNA-negative individuals.

Furthermore, anti-EBV antibody detection, consisting of Epstein-Barr nuclear antigen EBNA-IgG, EBV-VCA-IgG, EBV-VCA-IgA, EBV-VCA-IgM, and EBV-EA-IgG were conducted using chemiluminescence immunoassay in part of the subjects.

### Statistical analysis

All statistical analyses were performed using SPSS for Windows (version 21.0; IBM Corporation, Armonk, NY, USA) and Graphpad Prism 6. Follow-up was through September 2018. Progression-free survival (PFS) was calculated as the duration from initial diagnosis to the first time of progression, relapse from response, death or last follow-up. OS was defined as the time between diagnosis and death or last follow-up. The best cut-off values were determined by X-tile according to OS 12. Survival curves were carried out using the Kaplan-Meier method and statistical significance was assessed using the two-sided log-rank test. Mann-Whitney U test and Fisher exact test were respectively adopted to conduct correlation analysis between EBV DNA and continuous variables and categorical variables for unpaired samples. The Cox proportional hazards models were established for the estimation of factors in both univariate and multivariate analysis. Multivariate proportional hazards regression was performed with statistically significant factors from the univariate analysis. All statistical tests were two-sided, with *P*-values <0.05 defined significant.

## Results

### Patient characteristics

The baseline clinical and pathologic characteristics of 88 patients with MCL involved in this study at admission were presented in Table 1. Among the 88 patients, there were 69 males and 19 females with an M: F ratio of 3.6: 1, presenting a striking male predominance. The median age was 60 years (range, 28 to 81 years) and approximately 51% of the patients were over 60 years old. Thirteen (14.8%) patients had an ECOG score of more than 2. The majority of patients (94.3%) were at advanced stage (Ann Arbor stage III-IV) and 57 (64.8%) patients had BMI. Elevated LDH and β2-MG were shown in 19 (21.6%) and 60 (68.2%) patients, respectively. Thirty-eight (43.2%) patients were found suffering from B symptoms. The number of patients at low risk (sMIPI score 0-3), intermediate risk (sMIPI score 4-5) and high risk (sMIPI score 6-11) stratified by sMIPI were 40 (45.4%), 29 (33.0%) and 19 (21.6%), respectively.

Additionally, 27 (30.7%) patients were detected positive for EBV DNA and the median virus titer was 2.05×10^4^ copies/mL (range, 8.20×10^2^ to 4.14×10^5^ copies/mL). However, pretreatment results of anti-EBV antibody tests of 28 patients were obtained. Our results showed that 12 patients (42.9%) had serological evidence of prior EBV-infection (EBNA-IgG-positive and EBV-VCA-IgG-positive). Nine patients (32.1%) were at the late stage of infection (EBNA-IgG-positive, EBV-VCA-IgG-positive and EBV-EA-IgG-positive) while six patients (21.4%) were at the early stage of infection (EBV-VCA-IgG-positive, EBV-VCA-IgM-positive and EBV-EA-IgG-positive). Furthermore, one patient (3.6%) was found with the positive of EBNA-IgG, EBV-VCA-IgG, EBV-EA-IgG and EBV-VCA-IgM.

### Associations between EBV DNA and other baseline characteristics

Associations between EBV DNA load and other baseline characteristics were described in Table 1. In our cohort, a statistically evident dominance was identified that high level of EBV DNA copy number was more likely in males (*P*=0.047) and patients with high WBC (>6.7×10^9^/L) (*P*=0.037). Moreover, EBV DNA-positive patients seemed to take up a larger proportion in age >60yr group (*P*=0.066), intermediate- to high- risk group (*P*=0.077), indicating a tendency toward significance. With the use of X-tile plots, the cut-off point at 0.50×10^9^ monocyte cells/L yielded the highest difference in OS. Among the 42 patients whose monocyte cell counts exceeded the value, there were 17 (40.5%) patients in EBV DNA-positive group while 25 (59.5%) patients in EBV DNA-negative group. EBV DNA-positive patients seemed to take up a larger proportion in high AMC group (*P*=0.067). No significant correlations were observed between EBV DNA load and other factors.

### Clinical outcomes for MCL patients

Generally, we conducted efficacy assessment of each patient with the completion of treatment. Subjects received a median of six courses of immunochemotherapy (range 2-6). Median follow-up was 40 months (range, 9 to 120 months). Patients who achieved CR / CRu and PR accounted for 22.7% and 48.9%, respectively. Forty-three (48.9%) patients progressed and 28 (31.8%) died. For the entire cohort, the median PFS and OS were 32 months (range, 1-90 months) and 70 months (range, 2-116 months). The 3-, 5-year PFS rate was 38.0%±5.2% and 24.0%±6.3%, while the 3-, 5-year OS rate was 58.0%±5.6% and 48.0%±6.1%.

### Prognostic impacts of pretreatment EBV DNA in MCL

**Figure 1:**
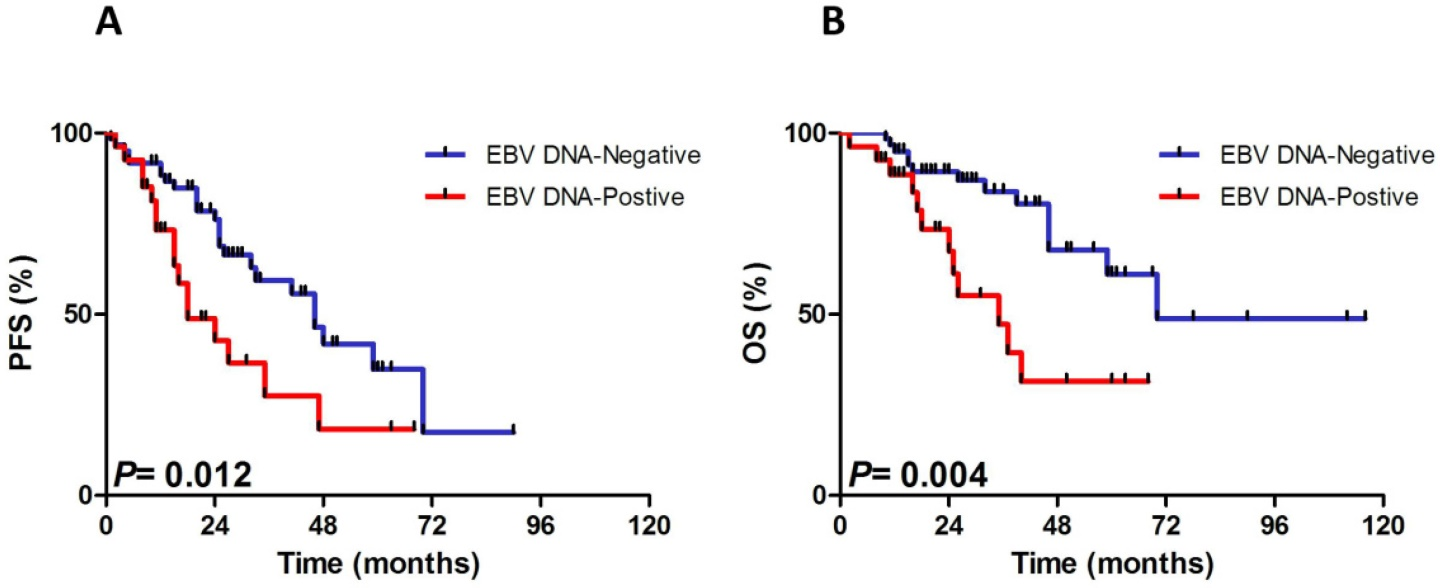
Progressive-free survival (PFS) and overall survival (OS) for 88 patients with the analysis of pretreatment Epstein-Barr virus (EBV) DNA status.

Not only did PFS estimation differ between the pretreatment EBV DNA-positive and EBV DNA-negative group (*P*=0.012), but also inferior OS was observed in the pretreatment EBV DNA-positive group than in the EBV DNA-negative group (*P*=0.004), which was shown in Fig. 1. The 3-year PFS and OS rate were 18.0%±8.4% and 31.0%±9.9% in EBV DNA-positive group vs. 46.0%±6.4% and 69.0%±6.7% in EBV DNA-negative group.

The univariate and multivariate Cox regression analyses of PFS and OS for potential risk predictors in MCL were summarized in Table 2 and Table 3. Univariate Cox regression analysis demonstrated that B symptoms (HR, 2.01; 95% CI, 1.07 to 3.78; *P*=0.029), LDH >upper limits of normal (ULN) (HR, 2.13; 95% CI, 1.07 to 4.21; *P*=0.030) and EBV DNA-positivity (HR, 2.20; 95% CI, 1.16 to 4.17; *P*=0.015) were related to inferior PFS. In addition, variables including male sex (HR, 8.62; 95% CI, 1.17 to 63.66; *P*=0.035), B symptoms (HR, 5.06; 95% CI, 2.08 to 12.32; *P*<0.001), LDH >ULN (HR, 2.53; 95% CI, 1.12 to 5.70; *P*=0.026), WBC >6.7×10^9^/L (HR, 2.69; 95% CI, 1.16 to 6.22; *P*=0.021), BMI (HR, 3.87; 95% CI, 1.32 to 11.33; *P*=0.014), high sMIPI score (HR, 1.75; 95% CI, 1.08 to 2.84; *P*=0.023) and EBV DNA-positivity (HR, 3.04; 95% CI, 1.38 to 6.69; *P*=0.006) had adverse effects on OS. Based on the univariate cox regression analysis results, sMIPI score and EBV DNA-positivity were put into multivariate analysis. As a result, multivariate Cox regression analysis revealed that EBV DNA-positivity was independent risk factor for both PFS (HR, 2.04; 95% CI, 1.07 to 3.92; *P*=0.031) and OS (HR, 2.68; 95% CI, 1.20 to 6.00; *P*=0.016).

### Subgroup evaluation of EBV DNA in MCL

**Figure 2:**
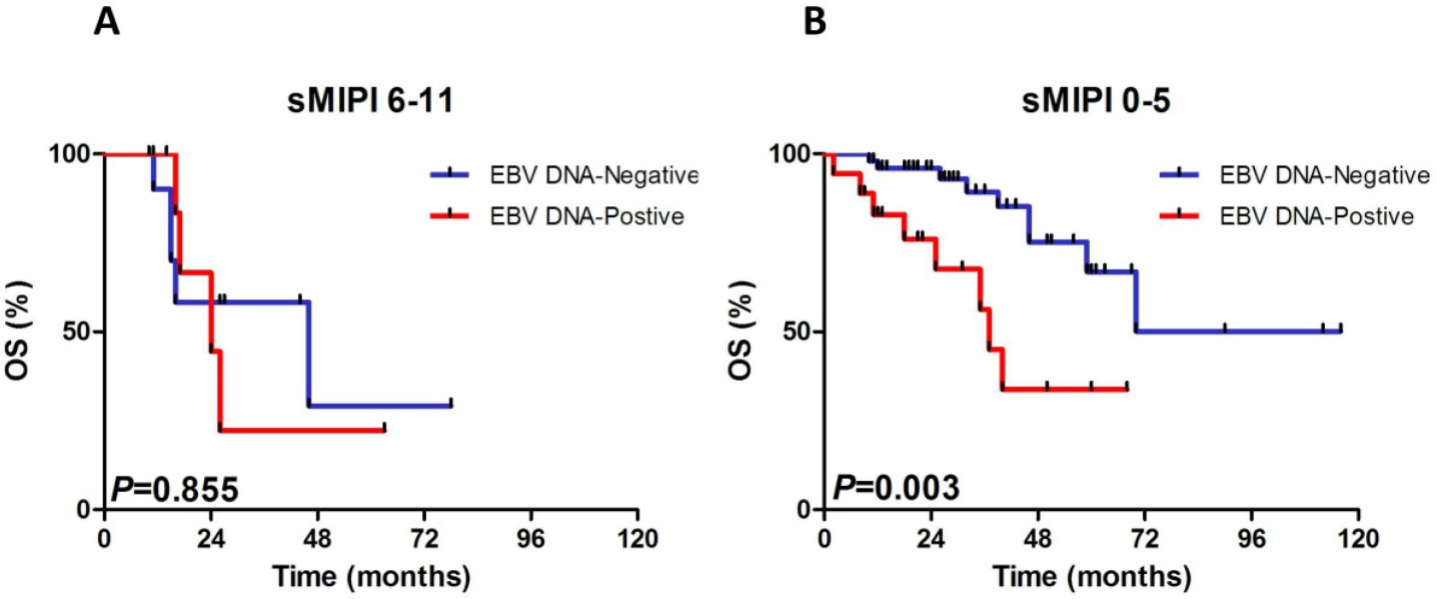
Kaplan-Meier curves of overall survival (OS) for different levels of EBV DNA load according to sMIPI score. sMIPI, simplified mantle cell lymphoma International Prognostic Index.

**Figure 3:**
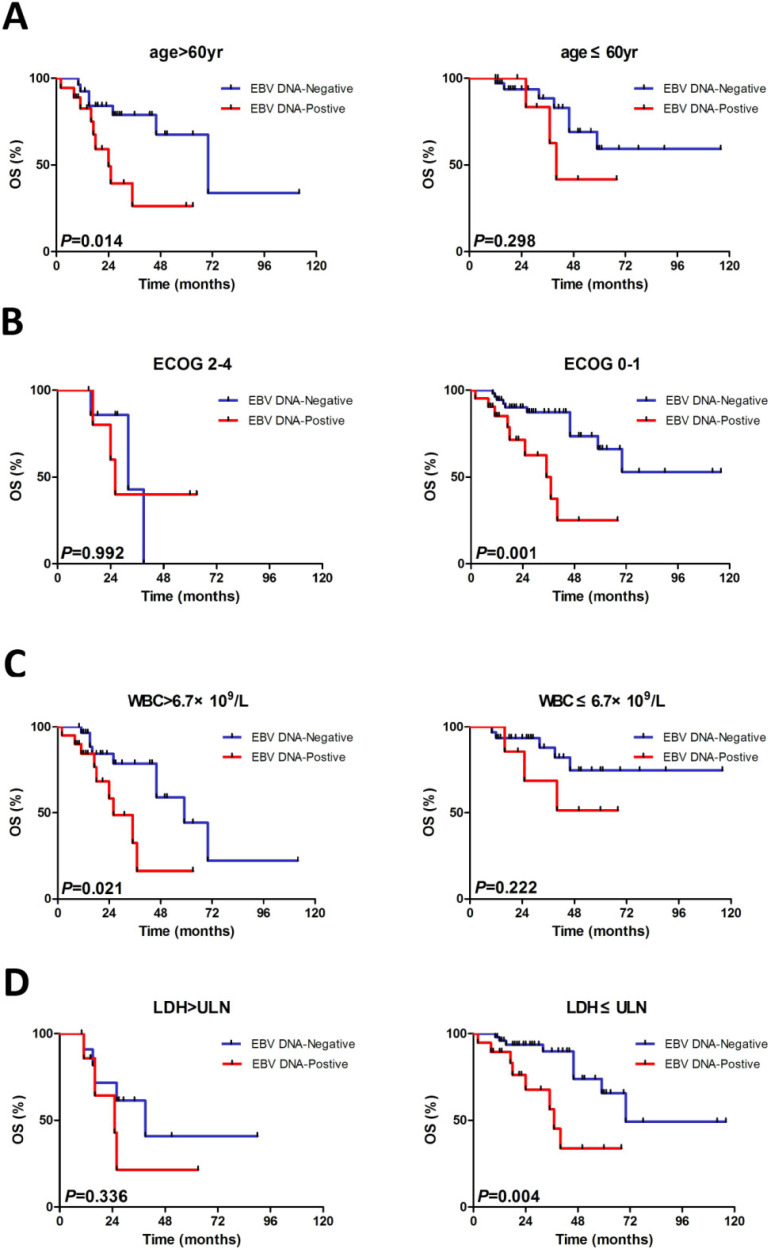
Kaplan-Meier curves of overall survival (OS) for different levels of EBV DNA load stratified by age (A), ECOG (B), WBC (C), LDH (D). ECOG, Eastern Cooperative Oncology Group; WBC, white blood cell counts; LDH, serum lactic dehydrogenase.

**Figure 4:**
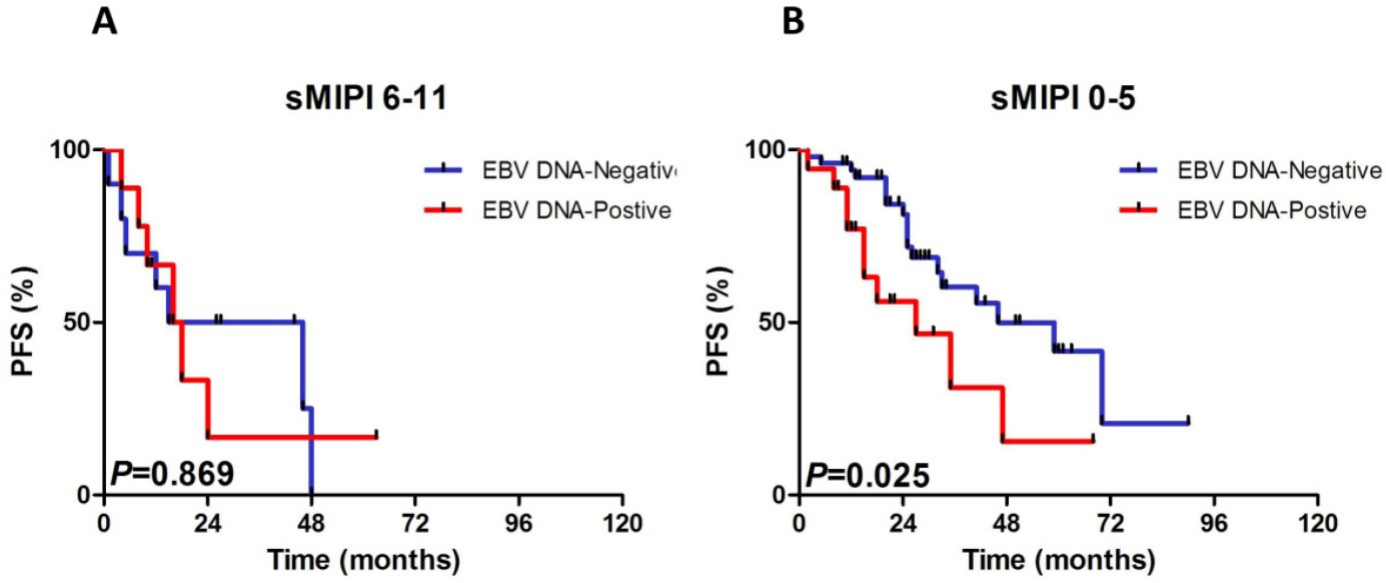
Kaplan-Meier curves of progressive-free survival (PFS) for different levels of EBV DNA load according to sMIPI score. sMIPI, simplified mantle cell lymphoma International Prognostic Index.

**Figure 5:**
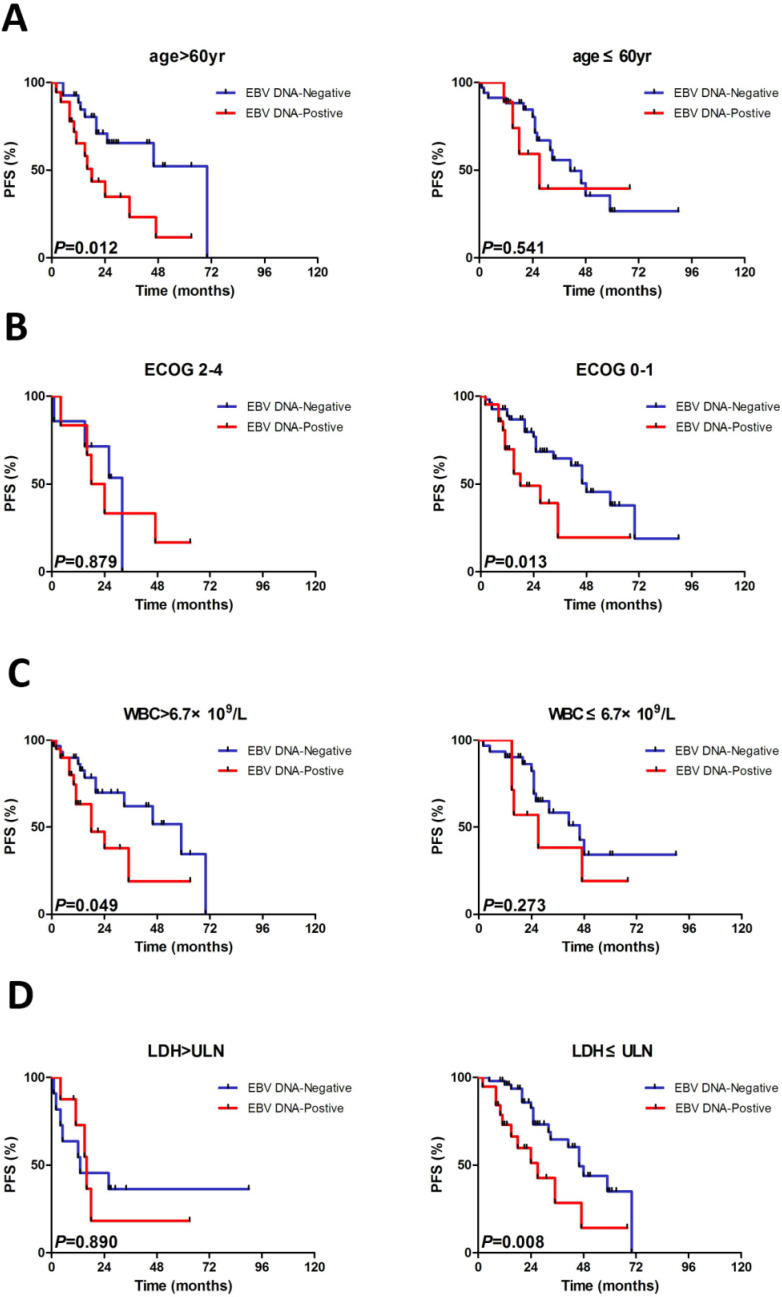
Kaplan-Meier curves of progressive-free survival (PFS) for different levels of EBV DNA load stratified by age (A), ECOG (B), WBC (C), LDH (D). ECOG, Eastern Cooperative Oncology Group; WBC, white blood cell counts; LDH, serum lactic dehydrogenase.

We constructed subgroup evaluations in order to accurately identify the influence of pretreatment EBV DNA load on PFS and OS combined with the risk parameters in sMIPI, as is illustrated in Fig. 2, Fig. 3, Fig. 4 and Fig. 5. The OS remained shorter in EBV DNA-positive subjects in subgroups such as sMIPI score 0-5 (*P*=0.003) (Fig. 2B), age >60yr (*P*=0.014) (Fig. 3A), ECOG 0-1 (*P*=0.001) (Fig. 3B), WBC >6.7×10^9^/L (*P*=0.021) (Fig. 3C), LDH ≤ULN (*P*=0.004) (Fig. 3D). Likewise, patients with EBV DNA positivity displayed inferior PFS in subgroups such as sMIPI score 0-5 (*P*=0.025) (Fig. 4B), age >60yr (*P*=0.012) (Fig. 5A), ECOG 0-1 (*P*=0.013) (Fig. 5B), WBC >6.7×10^9^/L (*P*=0.049) (Fig. 5C), LDH ≤ULN (*P*=0.008) (Fig. 5D).

### Dynamic changes in EBV DNA load

Among the 27 patients who were EBV DNA-positive at diagnosis, 17 patients had a sequential detection of EBV DNA copies during therapy and follow-up. EBV DNA copy numbers were undetectable in 12 patients who had good response to immunochemotherapy though four of them were dead at the end of follow-up deadline. Four patients had a significant increase in EBV DNA copy numbers when they experienced disease progression. One patient was assessed PR after the third cycle of immunochemotherapy regardless of elevated EBV DNA load at this time. However, this patient gave up the following treatment as a result of intolerance to immunochemotherapy due to infection.

## Discussion

In order to facilitate clinical decisions for MCL, prognostic indexes including sMIPI, Ki-67, TP53, serum β2-MG, chromosome karyotype have been assessed to enable risk-adapted treatment strategies 13-17. With more and more attention to EBV in recent years, EBV DNA load at initial diagnosis has been demonstrated to have an adverse prognostic impact on patients with several lymphomas 4-8. As is reported in literatures, EBV may activate B cells, stimulate their proliferation, and inhibit their apoptosis. Furthermore, EBV can express latent membrane protein 1 (LMP1), a latent protein expressed, leading to tumor angiogenesis by activating downstream signaling pathways and modulating the expression of cellular genes 18. However, to the best of our knowledge, it is the first time to interpret the relevance between EBV DNA and MCL though the specific intrinsic mechanism of EBV in MCL is unclear.

In this retrospective research, we checked the prognostic differences between EBV DNA-positive and EBV DNA-negative MCL in small scale and found that the pretreatment EBV DNA-positivity was an effective marker for predicting PFS and OS of MCL patients. Song et al revealed that lymphoma was at an increased risk among individuals with HBV. It was possible that HBV was harbored in non-liver cells, inducing local inflammation, which played a role in the development of cancer. One of the mechanisms of EBV might be the same as that of HBV 19. Besides, some demonstrated that high level of EBV DNA loads may represent a consequence of the deeper immunosuppression in CLL and AITL 20,21. As is demonstrated in AITL, Liang et al found that EBV DNA-positive patients were more associated with lower lymphocyte-monocyte ratio (LMR) 8. Actually, tumor microenvironment and host immunity are significantly associated with outcome in patients with MCL since AMC and absolute CD4+ T cell counts (ACD4C) have been confirmed to be significant predictors of unfavorable OS 22,23. In the present study, EBV DNA-positive patients tended to have high AMC, which reflected immune-deficient state for MCL patients to some extent, then adding to tumor aggressiveness, though statistical significance was not reached. Therefore, monitoring of AMC, T lymphocyte counts and natural killer cell counts as well as immunoglobulin level might be necessary for MCL patients. In addition, a significant correlation was noted between EBV DNA-positivity and male sex and WBC. Also, patients had high EBV DNA copies tended to be more likely in age >60yr group and intermediate- to high- risk group regardless of insignificant difference, in line with the standpoint that latently-EBV-infected B-cells induced sustained telomerase which was associated with an aggressive clinical behavior 24,25. These might account for the relationship between EBV DNA positivity and inferior OS in MCL patients.

In this current study, we only obtained 28 pretreatment results of anti-EBV antibody tests of MCL patients and failed to further explore the relevance between anti-EBV antibody and prognosis and clinical characteristics of MCL. However, according to 24-year experience at a single institution, Huang et al. provided convincing evidence that raised serum EBV EA-IgA and VCA-IgA levels were related to adverse ENKTL profile and correlated with poor treatment response, early relapse, and poor prognosis in patients with ENKTL 26. It served as a reminder that sufficient examinations including anti-EBV antibody tests should be regularly performed for our better understanding of MCL.

As we all known, the International Prognostic Index (IPI), as a traditional model for risk stratification of patients with aggressive non-Hodgkin lymphoma, was discarded for risk stratification of MCL as a result of its weak discriminatory, particularly for the lower risk patients. For better classification, we focused on subgroup analyses and found that the 3-, 5- year PFS and OS rate of EBV DNA-positive group were remarkably lower than EBV DNA-negative group (18.0%±8.4% and 31.0%±9.9% vs. 46.0%±6.4% and 69.0%±6.7%; 18.0%±0.0% and 31%±0.0% vs. 25.0%±9.0% and 55%±8.5%, respectively). Beyond that, EBV DNA-positive subjects were noted to have shorter PFS and OS in sMIPI score 0-5, ECOG 0-1 and LDH ≤ULN groups, indicating that the survivals of MCL patients with EBV DNA-positivity remained shorter even if he or she was classified as low-risk patients. In addition, patients with EBV DNA-positivity would have much worse PFS and OS if he or she was over 60yr and had high WBC.

Dynamic detection of EBV DNA changes is of equal important. In this cohort, a phenomenon raised our attention that patients who responded to treatment well were accompanied with decline of EBV DNA load while those who experienced progressed disease were with elevated EBV DNA copy numbers. Of interest, one of the patients was assessed PR after third cycle of treatment with elevated EBV DNA load at this time. Therefore, we come up with some speculations: (1) Whether EBV DNA load could be incorporated into prognostic index together with sMIPI worth further investigation since circulating EBV DNA level was added into the prognostic index for ENKTL (PINK-E); (2) Whether serial monitoring of EBV DNA quantitation after induction chemotherapy should be carried out regularly and formally based on protocol for all patients; (3) Whether appropriate antiviral therapy should be adopted to improve survivals of MCL patients with EBV DNA positivity since we don't have any anti-drug to fight against EBV at present.

It must be admitted that the current study has some limitations. Firstly, due to the retrospective nature, information bias exists. Secondly, on account of the low incidence of MCL, a small cohort of MCL patients was enrolled and the numbers of subjects in some subgroups were small, thus some of the results requiring validation in a larger cohort. Thirdly, Chinese population alone without other populations may limit our ability to discover more information. To sum up, pretreatment EBV DNA copy number was an important prognostic and monitoring marker for MCL patients which might be added into the further prognostic index based on large-scale, multi-central and multi-racial prospective studies for MCL patients.

## Figures and Tables

**Table 1:** Clinical characteristics with the analysis of pretreatment EBV DNA in 88 patients with mantle cell lymphoma

Characteristic	No. (%) (n=88)	EBV DNA-positivity (n=27)	EBV DNA-negativity (n=61)	*P*-value
Male sex	69 (78.4)	25 (92.6)	44 (72.1)	**0.047**
B symptoms	38 (43.2)	13 (48.1)	25 (41.0)	0.642
Age >60 yr	45 (51.1)	18 (66.7)	27 (44.3)	0.066
ECOG 2-4	13 (14.8)	6 (22.2)	7 (11.5)	0.207
Stage III-IV	83 (94.3)	27 (100.0)	56 (91.8)	0.318
LDH >ULN	19 (21.6)	8 (29.6)	11 (18.0)	0.265
WBC >6.7×10^9^/L	50 (56.8)	20 (74.1)	30 (49.2)	**0.037**
BMI	57 (64.8)	19 (70.4)	38 (62.3)	0.629
β2-MG >ULN	60 (68.2)	22 (81.5)	38 (62.3)	0.180
sMIPI score 4-11	48 (54.5)	18 (66.7)	30 (49.2)	0.077
AMC>0.5×10^9^/L	42 (47.7)	17 (63.0)	25 (41.0)	0.067
EBV DNA-positivity	27 (30.7)	-	-	-

ECOG, Eastern Cooperative Oncology Group; LDH, serum lactate dehydrogenase; ULN, upper limits of normal; WBC, white blood cells; BMI, bone marrow involvement; β2-MG, serum beta-2 microglobulin level; sMIPI, simplified mantle cell lymphoma International Prognostic Index; AMC, absolute monocyte counts; EBV, Epstein-Barr virus.

**Table 2:** Univariate Cox regression analysis of PFS and OS in 88 patients with mantle cell lymphoma

	Univariate analyses (PFS)		Univariate analyses (OS)	
Characteristic	HR (95% CI)	*P-*value	HR (95% CI)	*P*-value
Male sex	1.60 (0.70-3.63)	0.262	8.62(1.17-63.66)	**0.035**
B symptoms	2.01 (1.07-3.78)	**0.029**	5.06 (2.08-12.32)	**<0.001**
Age >60 yr	1.31 (0.71-2.42)	0.390	2.10 (0.95-4.63)	0.067
ECOG 2-4	1.73 (0.82-3.65)	0.148	2.05 (0.81-5.16)	0.128
LDH >ULN	2.13 (1.07-4.21)	**0.030**	2.53 (1.12-5.70)	**0.026**
WBC >6.7×10^9^/L	1.323 (0.71-2.46)	0.376	2.69 (1.16-6.22)	**0.021**
BMI	1.13 (0.58-2.20)	0.712	3.87 (1.32-11.33)	**0.014**
β2-MG >ULN	1.59 (0.73-3.47)	0.244	3.11 (0.92-10.45)	0.067
sMIPI score	1.38 (0.93-2.06)	0.111	1.75 (1.08-2.84)	**0.023**
EBV DNA-positivity	2.20(1.16-4.17)	**0.015**	3.04(1.38-6.69)	**0.006**

PFS, progression-free survival; OS, overall survival; HR, hazard ratio; CI, confidence interval; ECOG, Eastern Cooperative Oncology Group; LDH, serum lactate dehydrogenase; ULN, upper limits of normal; WBC, white blood cells; BMI, bone marrow involvement; β2-MG, serum beta-2 microglobulin level; sMIPI, simplified mantle cell lymphoma International Prognostic Index; EBV, Epstein-Barr virus.

**Table 3:** Multivariate Cox regression analysis of PFS and OS in 88 patients with mantle cell lymphoma

	Multivariate analyses (PFS)		Multivariate analyses (OS)	
Characteristic	HR (95% CI)	*P-*value	HR (95% CI)	*P*-value
sMIPI score	1.28 (0.85-1.91)	0.237	1.59 (0.98-2.59)	0.062
EBV DNA-positivity	2.04 (1.07-3.92)	**0.031**	2.68 (1.20-6.00)	**0.016**

sMIPI, simplified mantle cell lymphoma International Prognostic Index.
